# Mode Bifurcation on Contact Line Dynamics at Oil/Water Interface Depending on the Contact Line Length

**DOI:** 10.3389/fchem.2021.708633

**Published:** 2021-07-26

**Authors:** Daigo Yamamoto, Jumpei Maeno, Yuki Manabe, Yasunao Okamoto, Erika Nawa-Okita, Akihisa Shioi

**Affiliations:** ^1^ Department of Chemical Engineering and Materials Science, Doshisha University, Kyoto, Japan; ^2^ Department of Chemical Engineering, Osaka Prefecture University, Osaka, Japan

**Keywords:** contact line, interfacial energy, active matter, bifurcation, traveling-wave motion, up-and-down motion, oil/water interface

## Abstract

The motion of the contact line at the oil/water interface caused by chemical reactions is well known as a typical example of artificial active matter in the field of nonlinear science. When water (containing trimethylstearylammonium chloride) and nitrobenzene (containing iodide anion) phases are in contact, the regulated traveling-wave patterns appear along the inner wall of the glass container. In this study, we demonstrate a new dynamical mode of the contact line, an up-and-down motion, which becomes dominant with the decrease in the size of a glass tube, and the probability of occurrence is extremely high when the diameter of the glass tube is below 1 mm. A physicochemical model of the contact line motion that incorporates the spatiotemporal variation of the surfactant concentration on a glass surface is proposed, and its effect on the wettability of oil/water phases on the walls of the glass tubes is studied. The present model can reproduce the mode bifurcation of the dynamical motion depending on the inner diameter of the glass tubes.

## Introduction

Recently, active matter has become a common topic in mechanical engineering, biology, and nonlinear science (Ramaswamy, 2010; Marchetti et al., 2013; Nakata, 2019; Zhou et al., 2019; Gompper et al., 2020). Active matter exhibits autonomous motion by direct conversion of chemical into kinetic energy in the absence of an external force. It contains not only biological systems (e.g., microorganisms and living cells) but also non-living systems such as camphor boats (Nakata et al., 1997; Suematsu et al., 2010; Kitahata and Koyano, 2020), catalytic particles in solution (Paxton et al., 2004; Yamamoto and Shioi, 2015), and vesicles under a pH gradient (Nawa et al., 2013; Nawa et al., 2015). Since non-living active matter is easier to handle experimentally, a deeper understanding of active matter systems may be gained and used in engineering applications for innovative isothermal high-efficiency energy conversion. For this purpose, a physicochemical model of an artificial active matter system that exhibits biomimetic behavior needs to be constructed, using an experimental setup as simple as possible.

A typical example of an artificial active matter system is the motion of the oil/water interface (nitrobenzene/water) on a glass surface, which was reported in the 1970s (Dupeyrat and Nakache, 1978). Research on the motion of oil/water interfaces has been performed in nonlinear science, which is mainly divided into two types of experimental systems. One type is the motion of an oil droplet on a glass plate placed in the water phase (Sumino et al., 2005a; Sumino et al., 2005b; Sumino and Yoshikawa, 2008). Recently, we reported new applications of such motile oil droplets, such as in active transport (Goto et al., 2015) and synchronization (Kasai et al., 2020). The other type is the motion of the contact line between the inner wall of a cylindrical glass container and the oil/water interface (Kai et al., 1991; Shioi et al., 2003). The contact line forms a spatiotemporal wave that propagates in the circumferential direction of the container while maintaining its waveform (called traveling-wave motion). These motions are caused by repetitive adsorption/desorption of the surfactant and the consequent change in wettability of the oil/water phases on the glass surface (Shioi et al., 2008a; Shioi et al., 2008b; Sumino and Yoshikawa, 2008).

In this study, we focused on the motion of an oil/water contact line. By devising an experimental setup, we demonstrate a new dynamical mode instead of a traveling-wave motion, that is, an up-and-down motion. Furthermore, we propose a physicochemical model that considers the spatiotemporal variation in the adsorption/desorption process of the surfactants on the glass surface.

## Experiments

### Chemicals

Trimethylstearylammonium chloride (C_18_TAC, 98.0%) was purchased from Tokyo Chemical Industry Co., Ltd., Iodine (I_2_, 99.8%), potassium iodide (KI, 99.5%), and nitrobenzene (99.5%) were purchased from Wako Pure Chemical Industries, Ltd., All chemicals were used without further purification. A C_18_TAC aqueous solution (10 mM) was prepared as the water phase. Nitrobenzene saturated with KI was used as the oil phase, and 20 mM of I_2_ were dissolved in the oil phase.

### Observation of the Motion of Oil/Water Contact Line

**FIGURE 1:**
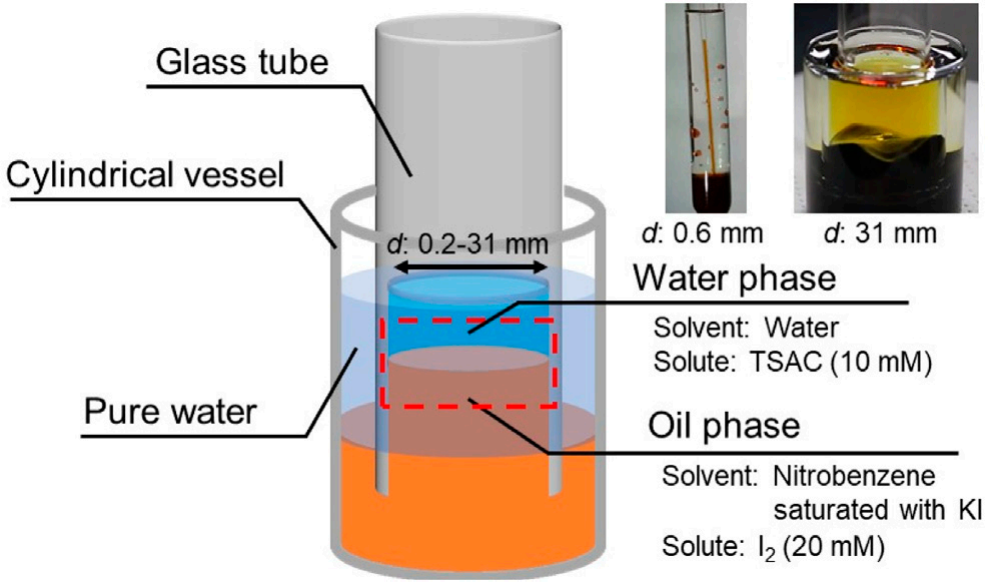
Experimental setup for observation of oil/water contact line motions.

In this study, we focused on the effect of the contact line length on its dynamical motion. A previous simple experimental setup with a glass container is not available because the volume preservation of the bottom oil phase significantly affects the contact line motion. This effect inhibits the contact line motion if the diameter of the glass container is considerably small. To eliminate the effect of volume preservation on the contact line motion, we used cylindrical glass tubes with open ends (open tube), instead of a glass container, to form a motile oil/water contact line (Figure 1).

Open tubes with various inner diameters (*d* = 0.2–31 mm) were tested. Each open tube was vertically inserted into the reservoir oil contained in a cylindrical vessel. Pure water was then poured into the space between the open tube and the cylindrical vessel to raise the surface level of the oil phase inside the open tube. Then, the water phase was poured into the open tube to form a motile oil/water contact line along the inner wall of the tube (see the red dashed area in Figure 1). The contact line motion was monitored using a digital single-lens reflex camera (Canon EOS Kiss X9) at a frame rate of 60 fps. All experiments were performed at room temperature (20–25°C).

## Experimental Results and Discussion

**FIGURE 2:**
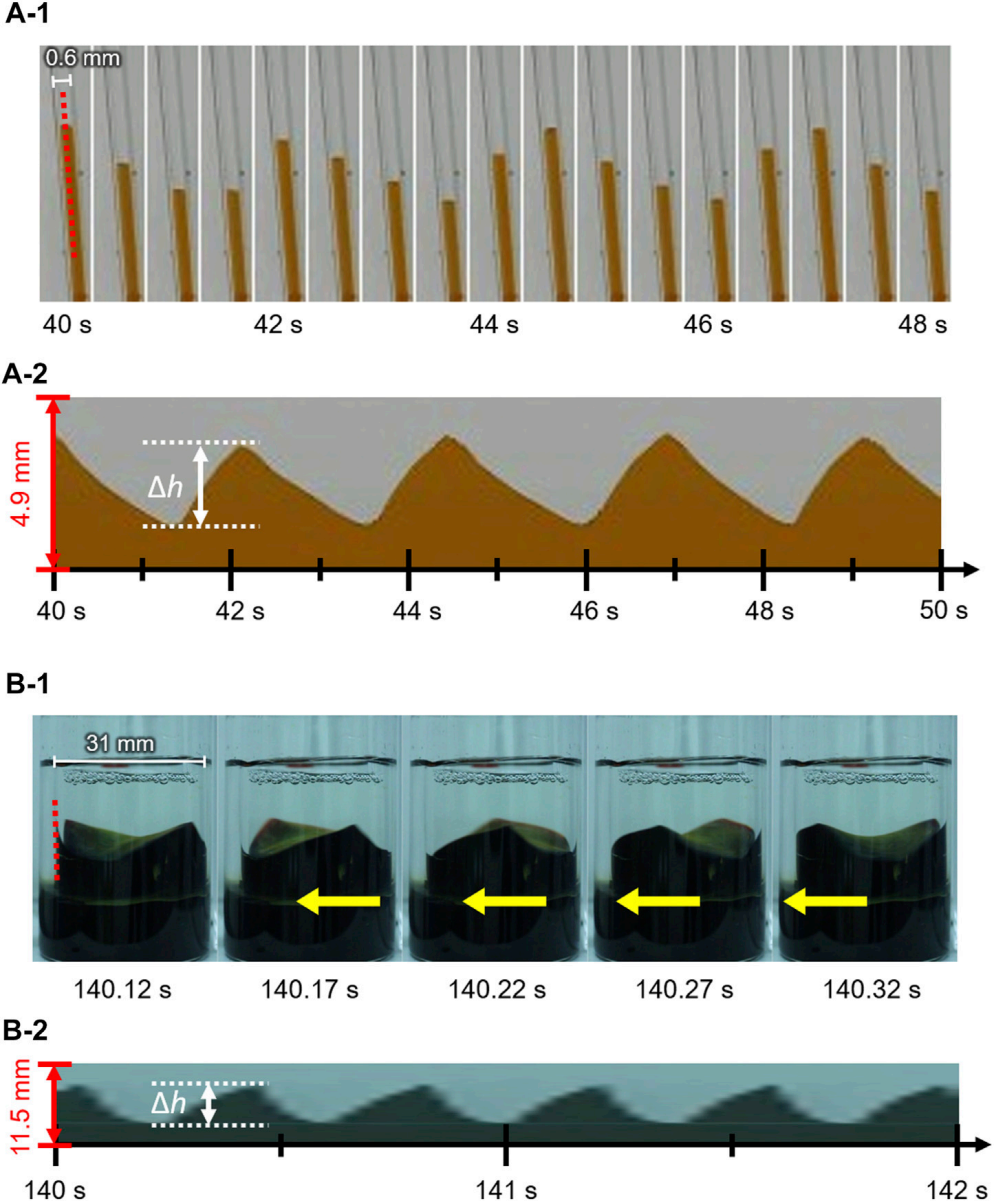
Snapshots and space–time plots of oil/water contact line motions in an open tube with an inner diameter of **(A)** 0.6 mm and **(B)** 31 mm. The contact line of **(A)** and **(B)** exhibits up-and-down motion and traveling-wave motion, respectively.

**FIGURE 3:**
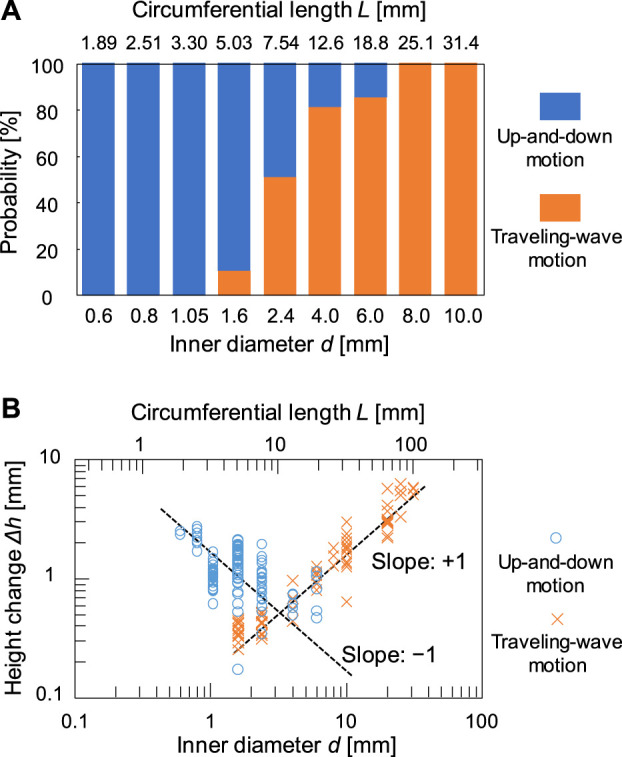
**(A)** Relationship between probability of occurrence of the two modes and the inner diameter *d* of tubes and **(B)** double logarithmic plot of height change Δ*h* and *d*.

Figures 2A–1 shows the contact line motion in an open tube with an inner diameter of 0.6 mm (= *d*) (circumferential length *L* (= π*d*) = 1.89 mm). The contact line moved up and down periodically for several minutes. (See Supplementary Video 1 for a smaller tube). Figures 2A–2 shows a space–time plot of the repetitive up-and-down motion along the red line shown in Figure 2A. The average of period *T* and the height change *∆h* for the up-and-down motion were estimated to be 2.5 s and 2.3 mm, respectively. The waveform of the space–time plot is asymmetric, which indicates that the rising speed of the contact line is faster than that of the falling contact line. Moreover, the waveform is convex for the rise and concave for the fall of the contact line. Hence, both rising and falling speeds decelerate as the contact line approaches the peak. Such an up-and-down motion was also observed for the glass tubes with intermediate inner diameters (*d* = 0.6–6.0 mm). However, a tube with a much larger inner diameter [*d* = 31 mm (*L* = 97.3 mm)] forms a regulated traveling wave (in this case, *∆h* is measured to 5.9 mm) (Figure 2B), as reported by several researchers including us (Dupeyrat and Nakache, 1978; Kai et al., 1991; Shioi et al., 2003). (See Supplementary Video 2 for a larger tube) These results demonstrate that the mode bifurcation on the contact line dynamics depends on the contact line length (the inner diameter of the glass tube). To investigate this mode bifurcation between the up-and-down and traveling-wave motions, we evaluated the ratio of each mode of open tubes with various inner diameters. The observed contact line motions are easily classified into the two modes in each experiment as follows. Briefly, when the traveling wave appears, the maximum and minimum of the height of the contact line is observed independent of time. Otherwise, the up-and-down motion keeps the almost same height at whole circumference in one snapshot. The experiments were repeated a minimum of four times for each inner diameter and the results obtained are shown in Figure 3A. In the tubes with diameter over 8.0 mm, only the traveling-wave motion was observed, while the up-and-down motion became dominant with a decrease in the diameter. In the tubes with intermediate diameters (*d* = 1.6–6.0 mm), either mode is observed in each experiment. (See Supplementary Video 3 for middle tubes). Once one mode appeared, it rarely changed to the other mode during the experiment. Actually, the larger tube tends to cause dynamical modes of longer duration (from several minutes to ten). In addition, the deviations of each Δ*h* is almost zero while the mode remains stable for several tens of cyclic periods. Such a bifurcation may be attributed to an initial fluctuation in the contact line. We confirmed that the two modes switched between each other by stimulating the motile contact line. To investigate the factors causing the bifurcation, the average *∆h* was measured for each trial (Figure 3B). As shown in Figure 3B, for the up-and-down motion, *∆h* decreases with an increase in the inner diameter. A plot of log (*∆h*) vs. log(*d*) for up-and-down motion gives a straight line with slope of roughly −1.0, although the distribution of *∆h* for the same inner diameter is large (probably due to surface condition of each glass tube we used). The slope of −1.0 can be explained by capillary action. When a capillary tube is dipped into liquid, the height *h* from the liquid interface is given by Jurin’s law (h∝γcos θ/d), where *γ* and *θ* denote the interfacial tension and contact angle on the contact line, respectively. In our experiment, the interfacial tension changed periodically by repetitive adsorption/desorption of surfactants (which corresponds to upward/downward motion of the contact line), while no appreciable change in the contact angle was observed. Assuming that the difference in the interfacial tension Δ*γ*_w/o_ between the maximum and minimum of the surface level is constant for various diameters, *∆h* is inversely proportional to the diameter of the tube (*∆h* $\propto \Delta \gamma_{\text{w}/\text{o}}\text{cos }\theta /d$). On the contrary, a larger inner diameter leads to a larger height change for the traveling-wave motion, as shown in Figure 3B. Thus, *∆h* of the two motions is comparable at the intermediate diameter (*d* = 2.4–6.0 mm), where both motions appear depending on the initial fluctuation of the contact line. We concluded that bifurcation can be determined by *∆h* of the two motions.

## Physicochemical Model for the Bifurcation

We have already proposed a mathematical model for traveling-wave motion (Shioi et al., 2008a; Shioi et al., 2008b). However, the previous model hardly considered any concrete physicochemical processes for the adsorption/desorption of surfactants. In this study, we propose a model that incorporates the spatiotemporal variation of the surfactant concentration on a glass surface and its effect on the wettability of oil/water phases on the walls of glass tubes. The equation for the contact line motion is expressed as follows:

$$m\frac{\partial^{2}h\left(t,x\right)}{\partial t^{2}}=-\mu \frac{\partial h\left(t,x\right)}{\partial t}+\Gamma \frac{\partial^{2}h\left(t,x\right)}{\partial x^{2}}-\chi \left[h\left(t,x\right)-h_{0}\right]+F\left(t,x\right)+\xi \left(t,x\right)$$ (1)

**FIGURE 4:**
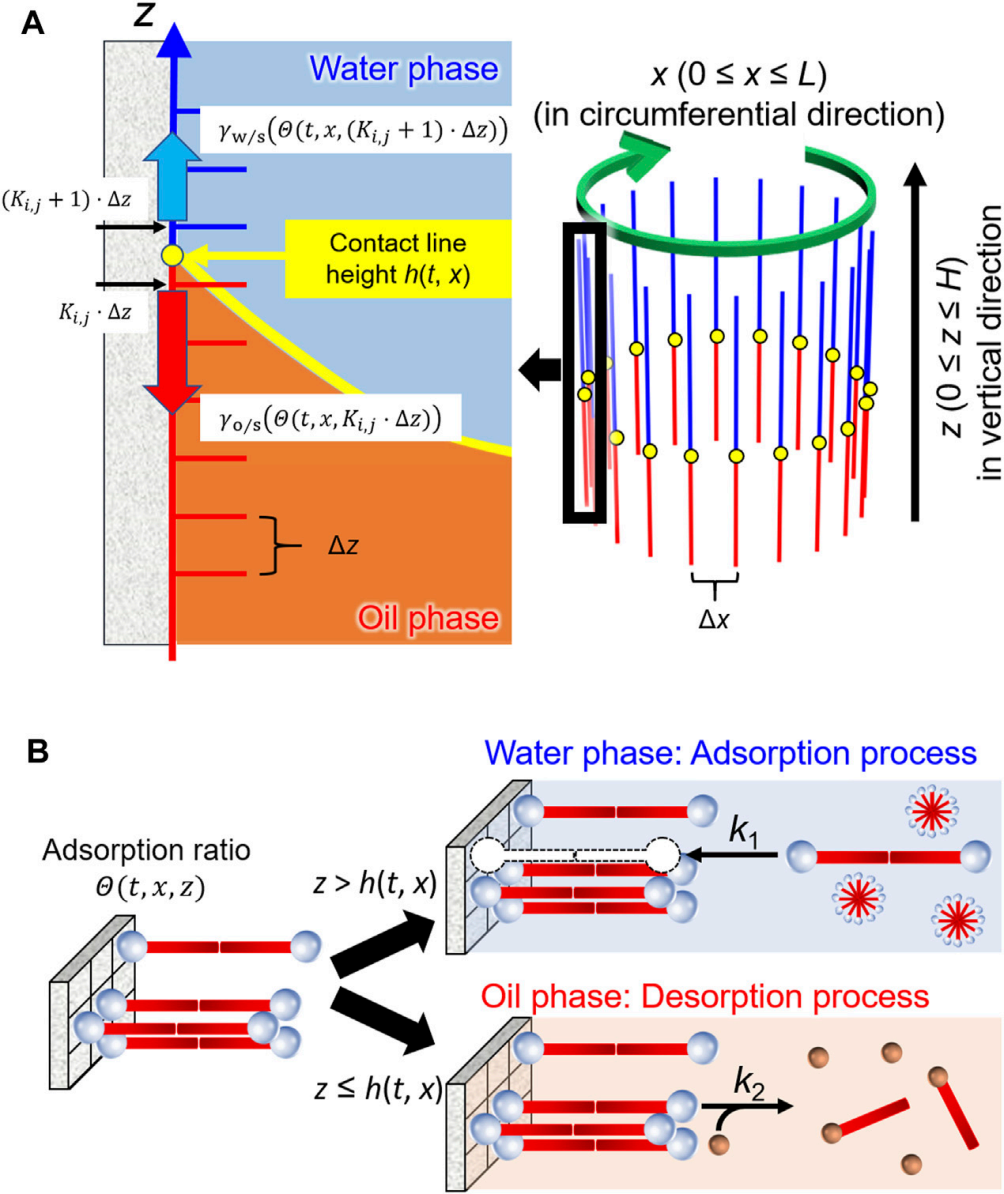
Schematic illustrations of the model for **(A)** the contact line motion along inner wall of a glass tube and **(B)** adsorption/desorption processes of the surfactants in water and oil phases.

As shown in Figure 4A, *h*(*t*, *x*) denotes the height of the contact line at the coordinate *x* (the circumferential displacement in our system) at time *t*. The first term (−*μ*∂*h*/∂*t*) on the right-hand side is the dissipation caused by the viscous drag force, where *μ* is the dumping coefficient. The second term (*Γ*∂^2^ *h*/∂*x* ^2^) works as the vibration control term, leading to a reduction in the roughness of the contact line shape due to interfacial tension (Shioi et al., 2008a). The third term (*χ*[*h*−*h*_0_]) is a restoring term owing to the density difference between the oil and water phases, which works as an elastic force that *h*(*t*, *x*) returns to the initial height of the contact line *h*_0_. The term is one of the most significant effects to reproduce the periodic motion. We confirmed in advance that the contact line maintains translation toward one direction when the term is negligible, which mimics our previous experimental results (Kasai et al., 2020). Here, *Γ* and *χ* denote proportional constants for each term. The fourth term (F(t,x)) is the driving force of the contact line motion caused by the wettability change of oil/water phases on the surface of the glass tube, which results from the repetitive adsorption/desorption of surfactants. The last term (ξ(t,x)) (time average: ξ(t,x) = 0) is a random noise acting on the oil/water interface for the Marangoni effect. To simplify the model, we assume that the Reynolds number of the contact line is so small that the inertia term on the left-hand side is negligible (*m*∂^2^ *z*/∂*t* ^2^ = 0) because the viscous force dominates the motion in such a millimeter-sized system.

In the present system, the water and oil phases are placed as layers at the upper and lower positions, respectively. Thus, the driving force caused by the solid–liquid interfacial energy is expressed as follows:

$$F\left(t,x\right)=\gamma_{\text{w}/\text{s}}{|}_{\Theta \left(t,x,h\right)}-\gamma_{\text{o}/\text{s}}{|}_{\Theta \left(t,x,h\right)}$$ (2)

$\gamma_{\text{w}/\text{s}}{|}_{\Theta \left(t,x,h\right)}$ and $\gamma_{\text{o}/\text{s}}{|}_{\Theta \left(t,x,h\right)}$ denote the solid–liquid interfacial energies of water/glass and oil/glass around the contact line (at *z* = *h*), where Θ(*t*, *x*, *z*) is the adsorption ratio of the surfactant at the vertical displacement z and at the circumferential displacement *x* of the glass surface (0 ≤ Θ(t,x,z) ≤ 1), as shown in Figure 4B.

Figure 4B illustrates the adsorption/desorption processes in this model. Most of the C_18_TAC dissolved in water forms micelles because the initial concentration is much higher than the CMC (critical micelle concentration = 0.3 mM). The resultant micelles were then adsorbed onto the glass surface as a bilayer (Atkin et al., 2003). Assuming that the micelle may be treated as a dimer of the cationic surfactant C_18_TA^+^, the absorption onto the site of the glass surface S, is described as follows:

$$\text{Water}\;\text{phase)}\;{\left({\text{C}}_{18}{\text{TA}}^{+}\right)}_{2}+\text{S}\to {\left({\text{C}}_{18}{\text{TA}}^{+}\right)}_{2}\cdot \text{S}$$ (3)

Here, we ignore the reverse reaction to simplify the model because the concentration of C_18_TAC is sufficiently high. In the oil phase, the desorption of the surfactant occurs because of the chemical reaction with oil-soluble anions, for example, the polyiodide anion I_*n*_^−^.

$$\text{Oil}\;\text{phase)}\;{\left({\text{C}}_{18}{\text{TA}}^{+}\right)}_{2}\cdot \text{S}+2{\text{I}}_{n}^{-}\to 2{\text{C}}_{18}\text{TA}\cdot {\text{I}}_{n}+\text{S}$$ (4)

From the above two equations, the reaction rate for the adsorption ratio can be expressed as:

$$\text{Water}\;\text{phase)}\;\frac{\text{d}\Theta \left(t,x,z\right)}{\text{d}t}=k_{1}\frac{C_{\text{A}0}}{2}\left(1-\Theta \left(t,x,z\right)\right)\left(\text{at}\;h\left(t,x\right)<z\leq H\right)$$ (5)

$$\text{Oil}\;\text{phase)}\;\frac{\text{d}\Theta \left(t,x,z\right)}{\text{d}t}=-k_{2}C_{10}^{2}\frac{C_{\text{A}0}}{2}\Theta \left(t,x,z\right)\;\left(\text{at}\;0\leq z\leq h\left(t,x\right)\right)$$ (6)

Here, *k*_1_ and *k*_2_ are the reaction rate constants for adsorption/desorption. *C*_A0_ is the C_18_TAC concentration in the water phase, which is divided by two to approximate the bilayer state. *C*_I0_ is the anion concentration in the oil phase. According to the Cassie model (Cassie, 1948), $\gamma_{\text{w}/\text{s}}\left(\Theta \left(t,x,h\right)\right)$ and $\gamma_{\text{o}/\text{s}}\left(\Theta \left(t,x,h\right)\right)$ around the contact line were estimated from the values of Θ(t,x,z) as follows:

$$\gamma_{w/s}|\Theta \left(t,x,h\right)=\Theta \left(t,x,h\right)\cdot \gamma_{w/s}\left(\Theta =1\right)+\left(1-\Theta \left(t,x,h\right)\right)\cdot \gamma_{w/s}\left(\Theta =0\right)$$ (7)

$$\gamma_{o/s}|\Theta \left(t,x,h\right)=\Theta \left(t,x,h\right)\cdot \gamma_{o/s}\left(\Theta =1\right)+\left(1-\Theta \left(t,x,h\right)\right)\cdot \gamma_{o/s}\left(\Theta =0\right)$$ (8)

$\gamma_{\text{w}/\text{s}}\left(\Theta =1\right)$, $\gamma_{\text{w}/\text{s}}\left(\Theta =0\right)$, $\gamma_{\text{o}/\text{s}}\left(\Theta =1\right)$, or $\gamma_{\text{o}/\text{s}}\left(\Theta =0\right)$ are the interfacial tensions of the water/glass or oil/glass phases at Θ = 1 or 0, respectively.

When this series of calculation processes (Equations 1, 2, 5, 6, 7, 8) is performed for all displacements *x* in the circumferential direction at time *t*, the time course of the contact line shape is obtained. Here, we preset the maximum height *H* for the contact line height (*h*). For the circumferential length *L* (= π*d*), a periodic boundary condition was applied.

The numerical calculation was performed using the finite-difference method. ∆*t*, ∆*x*, and ∆*z*, step width of *t*, *x,* and *z*, are 0.001, 0.01, and 0.001, respectively. Here, it is difficult to calculate the accurate value of Θ(t,x,h) at an arbitrary *h* because z in Equations 5, 6 is divided discretely in the numerical calculation. As *t*, *x*, and *z* can take *i* ∙ Δ*t* (*i* = 0, 1, 2, …, *i*_max_), *j* ∙ Δ*x* (*j* = 0, 1, 2, …, *j*_max_), and *k* ∙ Δ*z* (*k* = 0, 1, 2, …, *k*_max_), there exists an integer *K*_*i,j*_ that satisfies the following equation:

$$K_{i,j}\cdot \Delta z\leq h_{i,j}\left(=h\left(i\cdot \Delta t,j\cdot \Delta x\right)\right)<\left(K_{i,j}+1\right)\cdot \Delta z$$ (9)

**FIGURE 5:**
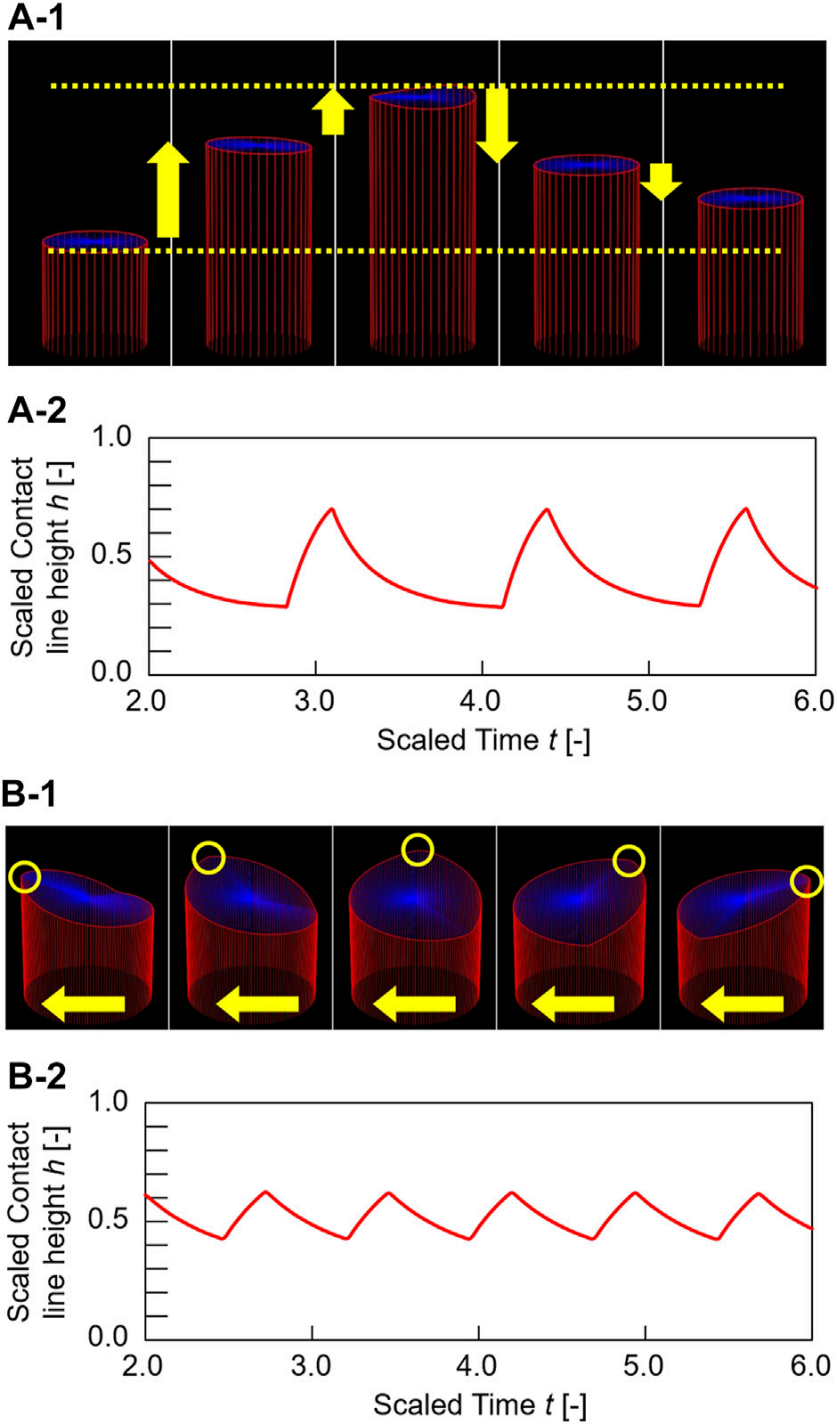
Calculation results. Three-dimensional graphs and time course of contact line height change *∆h* of the oil/water contact line motions for **(A)** *L* = 0.4 and **(B)** *L* = 1.5. The contact lines of **(A)** and **(B)** exhibit up-and-down motion and traveling-wave motion, respectively. Blue top surfaces in the three-dimensional graphs represent oil/water interfaces. The curved surfaces were drawn by connecting between the oil/water interfacial height at center *h*_c_(*t*) and the contact line height at arbitrary circumferential displacement. *h*_c_(*t*) was estimated by calculating the average of the contact line height at each time < *h*(*t*)> (h_c_(t) = ∫_0_^L^ h(t,x)dx/L).

In the calculation, $\gamma_{\text{w}/\text{s}}\left(\Theta \left(t,x,h\right)\right)$ and $\gamma_{\text{o}/\text{s}}\left(\Theta \left(t,x,h\right)\right)$ were approximated by $\gamma_{\text{w}/\text{s}}\left(\Theta \left(t,x,\left(K_{i,j}\;+1\right)\cdot \text{Δ}z\right)\right)$ and $\gamma_{\text{o}/\text{s}}\left(\Theta \left(t,x,K_{i,j}\cdot \text{Δ}z\right)\right)$, respectively, that is, Θ(t,x,h) in Equations 7, 8 are evaluated at *K*_*i,j*_ ∙ Δ*z*, and (*K*_*i,j*_ + 1) ∙ Δ*z* in the oil and water phases, respectively. This is shown in Figure 4A. Supplementary Note 1; Supplementary Figure 1 show procedure for calculating a physicochemical model of the contact line motion and a flowchart for the numerical solutions of the differential equations for the present model. The parameters used for our model are *μ* = 3.0, *Γ* = 0.12, *χ* = 15, ${\langle |\xi \left(t,x\right)|}^{2}\rangle$ = 0.03, *k*_1_ = 22, *k*_2_ = 0.3, *C*_A0_ = 10, *C*_I0_ =20, $\gamma_{\text{w}/\text{s}}\left(\Theta =1\right)$ = 9, $\gamma_{\text{w}/\text{s}}\left(\Theta =0\right)$ = 13, $\gamma_{\text{o}/\text{s}}\left(\Theta =1\right)$ = 2, $\gamma_{\text{o}/\text{s}}\left(\Theta =0\right)$ = 15, *z*_0_ = 0.5, and *H* = 1. At the initial conditions, Θ(t,x,z) at *t* = 0 is zero, which means that no surfactant is adsorbed on the glass surface. The initial height of the contact line *h*(*t*, *x*) at *t* = 0 is equal to *H* multiplied by random numbers between 0 and 1. This is because the shape of the initial contact line is complicated by the initial turbulence caused by pouring water on the oil phase. Figures 5A–1, B–1 show three-dimensional graphs of typical calculation results for *L* = 0.4 and 1.5. The contact lines exhibit up-and-down and traveling-wave motions, respectively, which are similar to the experimental results in Figures 2A–1, B–1. Figures 5A–2, B–2 show the time course of the contact line height h(t,x) at *x* = 0 for Figures 5A–1, B–1. The time variation shows an asymmetric pattern similar to the space–time plot in Figure 2.

**FIGURE 6:**
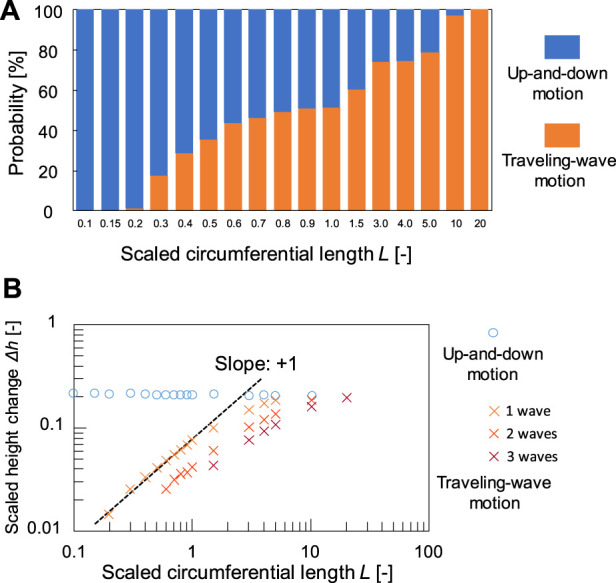
Calculation results. **(A)** Relationship between probability of occurrence of the two modes and scaled circumferential length *L* and **(B)** double logarithmic plot of height change Δ*h* and *L*. For the traveling-wave motion, the number of formed waves (*n*_TW_) is different for each trial. Thus, the plots of **(B)** are divided into three marks depending on *n*_TW_ for the traveling-wave motion. The plot for *n*_TW_ ≥ 4 was eliminated.

In the calculation, the dynamical mode is changed by the initial condition and the random force term, even in the same conditions (See Supplementary Video 4 for the simulation results). Once the contact line exhibits one mode, the mode remains stable for several cyclic periods. Therefore, we investigated the probability of the occurrence of the two modes by calculating 1,000 trials for each circumferential length (Figure 6). As shown in Figure 6, a remarkable mode bifurcation depending on *L* is observed. The up-and-down motion is dominant for *L* = 0.1–0.15, while the traveling-wave motion dominates for *L* = 20.0. In addition, a transition region exists for *L* = 0.2–10.0, which means that both motions are observed depending on the initial conditions and the random force term. This result reproduces the mode bifurcation qualitatively, similar to that shown in Figure 3A. These results demonstrated the validity of our physicochemical mechanism.

Finally, we compare the experimental and calculation results to investigate the bifurcation mechanism. The probability depending on the size is scaled appropriately and superposed in Supplementary Figure 2. A plot of probability vs. logarithm of the size in experiments has much larger slope than that in calculation, which means that the actual bifurcation is sharp depending on the inner diameter. This is because our model ignores the long-range effect that the height of the contact line is affected by that at the opposite side. The effect will become dominant for smaller tubes. In order to examine the contribution of the long-range effect, we investigated the height change Δ*h* for each circumferential length *L* in our model (Figure 6B). Because we neglect the interaction with the contact line at opposite side through capillary effect in our model, Δ*h* is almost constant regardless of the circumferential length for the up-and-down motion. The actual sharp bifurcation may be reproduced when the long-range effect is added in our model, whereas the calculation result predicts that the mode bifurcation can appear without the effect (for instance, by using flat plates with a different width).

On the other hand, a plot of log (Δ*h*) vs. log(*L*) for the traveling wave motion gives a straight line with slope of +1.0, which agrees with experimental results in Figure 3B. The reason why the larger tube gives the higher Δ*h* for the traveling-wave motion may be because the rate of adsorption/deadsorption is comparable to the traveling speed. We confirmed that traveling speed of the wave is almost the same regardless to the circumferential length for both experiment and simulation. Thus, when the same number of waves appears, the larger circumferential length leads to the longer period of the traveling-wave motion. This delay of arrival of the next wave affects time evolution of adsorption/deadsorption, which may result in the larger driving force deriving from the interfacial energy.

In the present stage, the detailed mechanism of the bifurcation is not elucidated completely because there are many parameters in our model. However, we need to note the contribution of elasticity of fringe (Gennes et al., 2010) and driving force, which correspond to second and fourth terms on the right-hand side in Equation 1. Both terms are derived from interfacial energy. We think that the balance between the amplitude of the two terms determines the bifurcation point. The physical kinetics of our system may be similar to other chemical systems such as size-depending bifurcation of Belousov–Zhabotinsky (BZ) patterns (Aihara and Yoshikawa, 2001), where the diffusion and growth rate of chemical wave.

## Conclusion

In this study, we demonstrated that the contact line motion at oil/water on a glass surface has two types of dynamical modes depending on the inner diameters of the glass tubes. That is, smaller inner diameters tend to originate up-and-down motion, while larger diameters tend to result in traveling-wave motion. Furthermore, to investigate the bifurcation mechanism, we proposed a physicochemical model of the contact line motion that considers the spatiotemporal variation of the adsorption/desorption process of the surfactants on the glass surface. The present model can reproduce the dynamics of both motion and mode bifurcation depending on the contact line length. However, our model has several parameters. The quantitative measurement of the reaction rate constants for adsorption/desorption and interfacial tension is desirable.

## Data Availability

The original contributions presented in the study are included in the article/Supplementary Material, further inquiries can be directed to the corresponding author.
