# Supplementary material for: Mode Bifurcation on Contact Line Dynamics at Oil/Water Interface Depending on the Contact Line Length
Source: Front Chem. 2021 Jul 26;9:708633. doi: 10.3389/fchem.2021.708633 (PMC8350764; doi:10.3389/fchem.2021.708633)
Supplement: Supplementary file 2 [file DataSheet1.PDF]

Supplementary Material for

Mode Bifurcation on Contact Line Dynamics at Oil/Water Interface Depending on the Contact Line Length

Daigo Yamamoto[1*], Jumpei Maeno[1], Yuki Manabe[1], Yasunao Okamoto[1], Erika Nawa-Okita[2], Akihisa Shioi[1]

[1] Department of Chemical Engineering and Materials Science, Doshisha University, Kyoto, Japan

[2] Department of Chemical Engineering, Osaka Prefecture University, Osaka, Japan

*** Correspondence:**
Corresponding Author
dyamamot@mail.doshisha.ac.jp

This Supplementary material includes:

Supplementary Note| Procedure for calculating a physicochemical model of the contact line motion.
Supplementary Figure 1| Flowchart for the numerical solutions of the differential equations for the present model.
Supplementary Figure 2| Scaling of the mode bifurcation depending on the size.
Supplementary Video 1| An up-and-down motion of the contact line in an open tube with an inner diameter of 0.6 mm.
Supplementary Video 2| Traveling-wave motion of the contact line in an open tube with an inner diameter of 31 mm.
Supplementary Video 3| Two types of contact line motions in an open tube with an inner diameter of 6.0 mm.
Supplementary Video 4| Simulation results of two types of contact line motions for L = 3.0.

Supplementary Note: Procedure for calculating a physicochemical model of the contact line motion.

The procedure used for calculating our model is described below.

The numerical calculation was performed using the finite-difference method. As t, x, and z can take $i \cdot \Delta t$ ($i = 0, 1, 2, \ldots, i_{max}$), $j \cdot \Delta x$ ($j = 0, 1, 2, \ldots, j_{max}$), and $k \cdot \Delta z$ ($k = 0, 1, 2, \ldots, k_{max}$), variables $f(t, x)$ and $g(t, x, z)$ can be expressed as $f_{i,j}$.and $g_{i,j,k}$, respectively. Equation 1 is transformed as follows.

$$0 = -\mu \frac{h_{i+1,j} - h_{i,j}}{\Delta t} + \Gamma \frac{h_{i,j+1} - 2h_{i,j} + h_{i,j-1}}{(\Delta x)^2} - \chi\left(h_{i,j} - h_0\right) + F_{i,j} + \xi_{i,j} \quad \text{(at low Reynolds number)}$$

Thus, $h_{i+1,j}$ is calculated using the following equation,

$$h_{i+1,j} = h_{i,j} + \frac{\Delta t}{\mu} \times \left\{\Gamma \frac{h_{i,j+1} - 2h_{i,j} + h_{i,j-1}}{(\Delta x)^2} - \chi\left(h_{i,j} - h_0\right) + F_{i,j} + \xi_{i,j}\right\} \quad \text{(Eq. S1)}$$

Similarly, Equations 5 and 6 is transformed as follows, respectively.

Water phase) $$\Theta_{i+1,j,k} = \Theta_{i,j,k} + k_1 \frac{C_{A0}}{2}\left(1 - \Theta_{i,j,k}\right) \times \Delta t \quad (\text{at } K_{i,j} < k \leq k_{max}) \quad \text{(Eq. S2)}$$

Oil phase) $$\Theta_{i+1,j,k} = \Theta_{i,j,k} - k_2 {C_{I0}}^2 \Theta_{i,j,k} \times \Delta t \quad (\text{at } 0 \leq k \leq K_{i,j}) \quad \text{(Eq. S3)}$$

Furthermore, Equations 7, 8, and 2 is transformed as follows, respectively.

$$\gamma_{w/s}(\Theta_{i,j,k}) = \Theta_{i,j,k} \cdot \gamma_{w/s}(\Theta = 1) + \left(1 - \Theta_{i,j,k}\right) \cdot \gamma_{w/s}(\Theta = 0) \quad \text{(Eq. S4)}$$

$$\gamma_{o/s}(\Theta_{i,j,k}) = \Theta_{i,j,k} \cdot \gamma_{o/s}(\Theta = 1) + \left(1 - \Theta_{i,j,k}\right) \cdot \gamma_{o/s}(\Theta = 0) \quad \text{(Eq. S5)}$$

$$F_{i,j} = \gamma_{w/s}(\Theta_{i,j,k}) - \gamma_{o/s}(\Theta_{i,j,k}) \quad \text{(Eq. S6)}$$

Thus, we obtain time course of contact line height change Δh of the oil/water contact line motions according to the flowchart as shown in Figure S1.

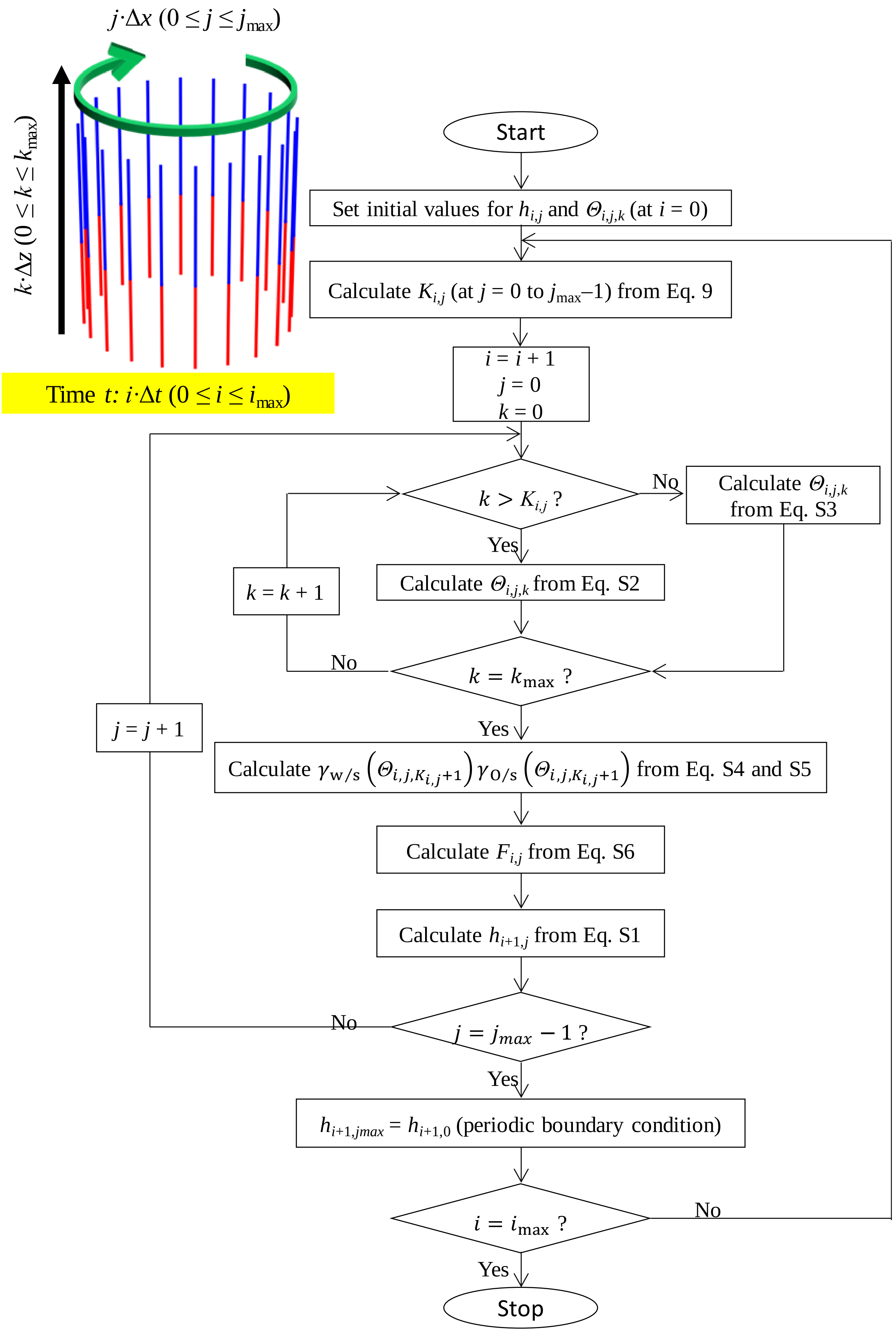

Supplementary Figure 1: Flowchart for the numerical solutions of differential equations for the present model.

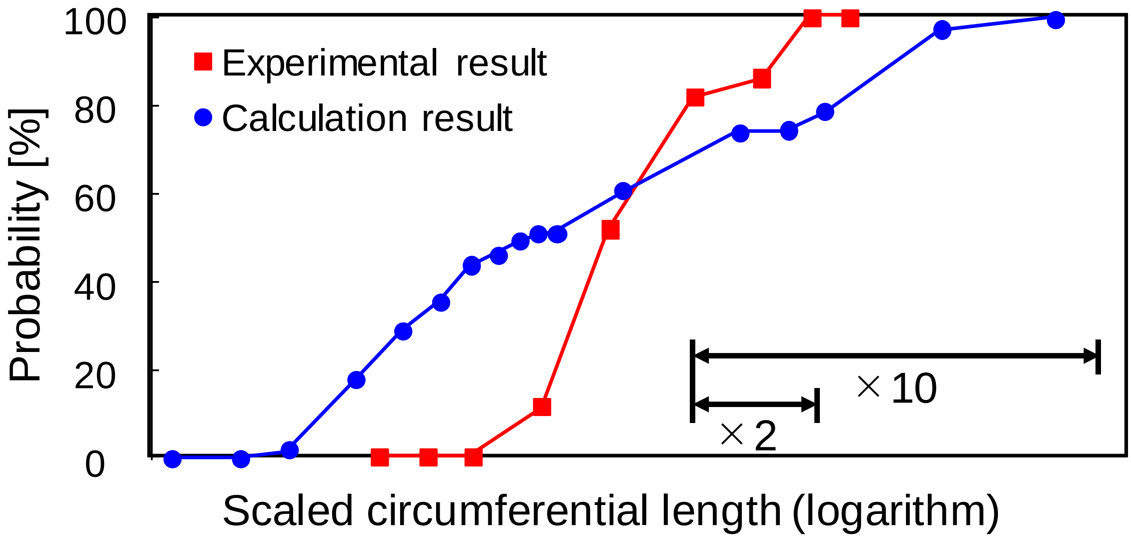

Supplementary Figure 2: Scaling of the mode bifurcation depending on the size.

(a plot of probability vs. logarithm of the size)
